# Basic Analysis of the Cerebrospinal Fluid: An Important Framework for Laboratory Diagnostics of the Impairment of the Central Nervous System

**DOI:** 10.3390/cimb44080251

**Published:** 2022-08-14

**Authors:** Petr Kelbich, Karel Hrach, Jan Spicka, Petr Vachata, Tomas Radovnicky, Eva Hanuljakova, Jan Krejsek

**Affiliations:** 1 Department of Biomedicine and Laboratory Diagnostics, University J.E. Purkinje and Masaryk Hospital, 401 13 Usti nad Labem, Czech Republic; 2 Department of Clinical Immunology and Allergology, Faculty of Medicine and University Hospital, Charles University, 500 03 Hradec Kralove, Czech Republic; 3 Laboratory for Cerebrospinal Fluid, Neuroimmunology, Pathology and Special Diagnostics Topelex, 190 00 Prague, Czech Republic; 4 Faculty of Health Studies, University J.E. Purkinje, 400 96 Usti nad Labem, Czech Republic; 5 Department of Neurosurgery, University J.E. Purkinje and Masaryk Hospital, 401 13 Usti nad Labem, Czech Republic; 6 Department of Neurosurgery, Faculty of Medicine and University Hospital, Charles University, 301 00 Pilsen, Czech Republic

**Keywords:** cerebrospinal fluid, cytological-energy analysis, coefficient of energy balance, blood-cerebrospinal fluid barrier, blood-brain barrier, aspartate aminotransferase

## Abstract

Laboratory analysis of basic cerebrospinal fluid (CSF) parameters is considered as essential for any CSF evaluation. It can provide rapidly very valuable information about the status of the central nervous system (CNS). Our retrospective study evaluated parameters of basic CSF analysis in cases of either infectious or non-infectious CNS involvement. Neutrophils are effector cells of innate immunity. Predominance of neutrophils was found in 98.2% of patients with purulent inflammation in CNS. Lymphocytes are cellular substrate of adaptive immunity. We found their predominance in 94.8% of patients with multiple sclerosis (MS), 66.7% of patients with tick-borne encephalitis (TBE), 92.2% of patients with neuroborreliosis, 83.3% of patients with inflammatory response with oxidative burst of macrophages in CNS and 75.0% of patients with malignant infiltration of meninges (MIM). The simultaneous assessment of aerobic and anaerobic metabolism in CSF using the coefficient of energy balance (KEB) allows us to specify the type of inflammation in CNS. We found predominantly aerobic metabolism (KEB > 28.0) in 100.0% CSF of patients with normal CSF findings and in 92.8% CSF of patients with MS. Predominant faintly anaerobic metabolism (28.0 > KEB > 20.0) in CSF was found in 71.8% patients with TBE and in 64.7% patients with neuroborreliosis. Strong anaerobic metabolism (KEB < 10.0) was found in the CSF of 99.1% patients with purulent inflammation, 100.0% patients with inflammatory response with oxidative burst of macrophages and in 80.6% patients with MIM. Joint evaluation of basic CSF parameters provides sufficient information about the immune response in the CSF compartment for rapid and reliable diagnosis of CNS involvement.

## 1. Introduction

Basic cerebrospinal fluid (CSF) analysis is a very important approach to quickly assess the current state of the central nervous system (CNS). Despite this fact, basic CSF examination is often underestimated. The aim of this study is to present our scheme of basic CSF examination, to show the interpretation of results in several subgroups of patients with different CNS involvement and to stimulate interest in this important part of CSF analysis.

Our basic CSF examination consists of a simultaneous assessment of the blood-cerebrospinal fluid barrier (BCB) permeability (see 1.1.), cytological composition (see 1.2.), energy ratios (see 1.2.) and detection of CNS tissue damage (see 1.3.). Measurement of total CSF protein concentration or albumin quotient is used to assess BCB permeability. Cytological analysis provides the essential information, especially addressing the presence of immunocompetent cells in CSF, possibly also the presence of tumor cells, signs of tissue damage, bleeding, presence of microbial pathogens, etc. Energy parameters, i.e., the simultaneous assessment of immunocompetent cells and KEB values in CSF is called cytological-energy analysis and allows us to determine intensity and the type of local inflammatory response in the CNS. CNS tissue damage at the level of baseline CSF examination is evidenced by aspartate aminotransferase (AST) catalytic activity determination.

### 1.1. Cerebrospinal Fluid Production and Blood-Cerebrospinal Fluid Barrier Permeability (Figure 1)

**Figure 1:**
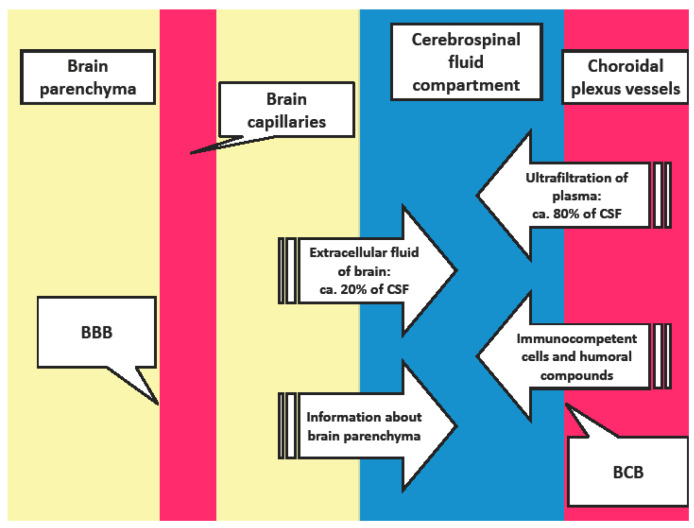
Schematic representation of CSF production. BCB: Blood-Cerebrospinal Fluid Barrier; BBB: Blood-Brain Barrier.

Approximately 80% of cerebrospinal fluid (CSF) is produced by ultrafiltration of blood plasma through the endothelium of the choroid plexus vessels. This structure is called the blood-cerebrospinal fluid barrier (BCB) and regulates the flow of immune system components into the CSF. The absence of pathological processes in the CSF is accompanied by the influx of a limited number of immunocompetent cells and a low concentration of proteins. In contrast, pathological processes in the CSF are associated with an increased number of cells and changes in the concentration of humoral components in the CSF. This condition is usually referred to as “increased BCB permeability”. It is evidenced as elevated concentration of total protein in CSF or albumin quotient (Qalb. = albumin in CSF/albumin in blood) (Figure 1) [1,2,3,4,5].

The brain parenchyma is highly vascularized. The endothelial cells of the brain capillaries are one from the key components of blood-brain barrier (BBB), which significantly influences the composition of the extracellular fluid in the brain. After crossing the ventricular wall, the fluid replenishes the remaining approximately 20% volume of the CSF. Therefore, CSF is thus an important source of information about the physiology or pathophysiology of the brain parenchyma (Figure 1) [2,5,6,7].

### 1.2. Cytological-Energy Analysis of the CSF

The evaluation of cytological and energy parameters in the CSF compartment is performed in two steps. The first is to determine the number and composition of immunocompetent cells in the CSF. The second is to determine the level of their activation by examining energy parameters in the CSF. To determine energy parameters, we have recently proposed so-called coefficient of energy balance (KEB; in Czech “Koeficient energetické bilance”). KEB is calculated using the molar concentrations of glucose and lactate in CSF and calculate as followed:

$$\mathrm{KEB}=38-18\frac{\left[\mathrm{lactate}\right]}{\left[\mathrm{glucose}\right]}$$

KEB is defined as the theoretical average number of adenosine triphosphate (ATP) molecules that are produced from one glucose molecule under the appropriate energy conditions in the CSF compartment. Activation of immunocompetent cells correlates with an increase in glucose and oxygen consumption and the development of anaerobic metabolism in CSF. The decrease in ATP production is reflected by a decrease in KEB values [8,9,10,11,12,13,14,15,16,17].

Based on the KEB values, we stratified our cases as follows:

- 28.0 to 38.0: aerobic metabolism in the CSF.
- 20.0 to 28.0: slight anaerobic metabolism in the CSF.
- 10.0 to 20.0: moderate anaerobic metabolism in the CSF.
- <10.0: strong anaerobic metabolism in the CSF.

### 1.3. Detection of CNS Tissue Injury

We consider aspartate aminotransferase (AST) catalytic activity in the CSF to be a readily available, easily measurable and inexpensive parameter for reliable assessment of CNS injury. This enzyme is present in all nucleated cells. Cellular damage is characterized by release of AST with subsequent elevation of AST activity in body fluids including CSF [8,18].

### 1.4. Absence of Pathology in the CSF

The absence of a pathological process in the CNS is characterized by no significant immune response in the CSF. There is the only basal immune surveillance there. The cellular and humoral components of the immune system in the CSF are at a basic level. The maximum leukocyte count in CSF is 4 cells/1 µL with predominance of resting lymphocytes (about 70%) and a minority of resting monocytes (about 30%). The low concentration of total protein in the CSF (<430.0 mg/L) indicates normal permeability of the BCB or the absence of any disturbance in the CSF circulation. Energy metabolism in CSF is aerobic with KEB values above 28.0. AST catalytic activity below 18.0 IU/L is not indicating tissue destruction in the CNS. No signs of hemorrhage are detected by CSF cytology [8,10,12,14,18,19,20].

### 1.5. Inflammatory Response in the CSF Compartment

The inflammatory response in the CSF compartment is followed by mobilization of cellular and humoral components into the CSF. The consequence of increased BCB permeability is increase in total protein concentration in CSF. The number of cells in the CSF is increased and their composition is reflecting the type of inflammatory response. Immunocompetent cells are activated, and more energy is required. Glucose and oxygen consumption is increased in this way. The concentration of glucose in the CSF is decreased. There is the switch from aerobic glucose metabolism to anaerobic one. Energy production in the form of adenosine triphosphate (ATP) molecules is decreased. This process is revealed as decrease in KEB values. In addition, local inflammation in the CNS may be associated with tissue destruction resulting in increased AST catalytic activity in the CSF [8,10,12,14,15,18,19,20].

### 1.6. Infectious Impairment of the CNS

Precise determination of the nature of the inflammatory reaction in the CSF can define the spectrum of causes of CNS pathologies.

Neutrophils represent the major population of phagocytic cells and are the final effector cells of innate immunity, with a primary role to clear extracellular pathogens [16]. The progression of purulent inflammation is based on the neutrophils oxidative burst with the production of reactive oxygen species (ROS). This is responsible for enhanced oxygen consumption. In sum, extensive accumulation of neutrophils and strong anaerobic metabolism in the CSF (KEB < 10.0) together with increased BCB permeability reveal purulent inflammation induced by the presence of extracellular bacteria in the CNS [8,9,10,12,14,15,17,21,22,23,24,25,26,27,28].

Increased BCB permeability, pleiocytosis with lymphocytes predominance, and aerobic or faintly anaerobic metabolism in the CSF compartment (28.0 > KEB > 20.0) usually represent the presence of serous inflammatory response in the CSF induced by either virus or spirochetes present in the CNS [10,12,14,19,29,30,31,32,33,34,35,36,37,38,39,40,41].

Very similar findings in CSF corresponding to mononuclear or lymphocytic pleiocytosis, hyperproteinorhachia, hypoglycorhachia and hyperlactatorhachia have been described in patients with neurotuberculosis, neurolisteriosis and cryptococcal meningitis [10,19,32,42,43,44,45,46,47,48,49]. In contrary, Bicanic and Harrison (2004) reported normal CSF white cell counts in HIV-associated cryptococcal meningitis, probably reflecting inability to mount protective immune response in these patients [49].

### 1.7. Inflammatory Response in the CNS to Non Infectious Stimuli

There are also numerous non-infectious causes of inflammatory response in CSF, such as autoimmunity, injury, hemorrhage, ischemia, tumors and neurodegenerative disorders [4,6,9,10,50,51,52,53,54,55].

### 1.8. Multiple Sclerosis

Multiple sclerosis (MS) is an autoimmune immunopathological disease affecting primarily white matter of the brain and spinal cord. Immunopathological inflammation targets myeline sheets of neurons thus impairing nerve signal transduction with ultimate axonal loss. Other CNS structures, including oligodendrocytes, are also targeted. Blood-brain barrier (BBB) contributes significantly to the pathogenesis of multiple sclerosis. It is a gateway for autoreactive lymphocytes entry into brain parenchyma. Basic analysis of CSF of patients with multiple sclerosis usually shows only subtle nonspecific changes. Total protein concentrations are often within normal limits, leukocyte counts are low or only slightly elevated, CSF energy ratios are usually insignificant, and CNS tissue destruction is not apparent. Lymphocytic oligocytosis or slight lymphocytic pleiocytosis with predominance of activated lymphocytes and the presence of plasmocytes in the CSF are frequently found. The gold standard of laboratory analyses in multiple sclerosis is still detection of intrathecal oligoclonal immunoglobulin synthesis by isoelectric focusing of CSF and blood [4,10,11,54,55,56,57,58]. However, this approach is not used in our present study.

### 1.9. Malignant Infiltration of Meninges

Cytological analysis of CSF plays a key role in the detection of malignant infiltration of the brain meninges (MIM). Deep analysis of inflammatory parameters can identify the presence of malignancy in CNS even if tumor cells in the CSF cytology are absent. Elevated proteins, pleiocytosis with predominance of lymphocytes and hypoglycorrhachia are characteristic in CSF patients with malignant infiltration of meninges [51,53,59,60,61].

## 2. Material and Methods

This retrospective study was approved by the local Ethics Committee of the Masaryk Hospital Usti nad Labem, Czech Republic (reference number: 305/19). No informed consent was required for this study as this work did not involve any human experiment. All patient records and information were anonymized and deidentified.

We performed a basic analysis of 524 cerebrospinal fluid samples evaluated as normal serving as controls for this study (Normal). In total, 304 CSF samples from patients with multiple sclerosis (MS), 39 CSF samples from patients with tick-borne encephalitis (TBE), 51 CSF samples from patients with central neuroborreliosis (NB), 113 CSF samples from patients with purulent inflammation (P) in the CNS induced by extracellular bacteria, and 31 CSF samples from patients with malignant infiltration of meninges (MIM) were enrolled to this study. We separately evaluated 6 CSF samples taken from 1 patient with cryptococcal meningitis, 1 patient with neurotuberculosis, 1 patient with neurolisteriosis and 3 patients with neuroborreliosis. These CSF samples were evaluated as “serous” inflammation in terms of cytological analysis and “purulent” in terms of energy analysis (Table 1).

### 2.1. Determination of the Blood-Cerebrospinal Fluid Barrier Permeability

BCB permeability was assessed using only cerebrospinal fluid total protein concentrations. We did not evaluate the albumin quotient because in some emergency cases cerebrospinal fluid samples only without blood samples were analyzed.

Cerebrospinal fluid samples were centrifuged, and the mass concentration of total protein was determined by the turbidimetry method with bensetonium chloride on a Cobas 6000 analyzer (Roche Diagnostics, Basel, Switzerland).

### 2.2. Cytological-Energy Analysis of CSF

The samples of CSF were collected into tubes without anticoagulants and immediately transported to our clinical laboratory. The total number of elements in these samples was enumerated using a Fuchs-Rosenthal chamber under the optical microscope. Cytological smear using cytocentrifuge method was prepared immediately after receiving the sample in all cases. Permanent cytological smears were stained using Hemacolor (Merck Co., Gernsheim, Germany). Microscopic analyses to determine cellular composition of CSF were performed by trained laboratory personal using Olympus BX40 microscope (Olympus, Tokyo, Japan).

Another aliquot of the samples was centrifuged and the molar concentrations of glucose using the hexokinase method and lactate using the lactate-oxidase and peroxidase method on a Cobas 6000 analyzer (Roche Diagnostics, Basel, Switzerland) were determined.

KEB values were calculated for all samples, including rare cases with very low glucose concentrations below the measurement limit (=0.11 mmol/L). Glucose concentration of 0.11 mmol/L was used to calculate KEB values in all these anaerobic cases.

### 2.3. Assessment of CNS Tissue Injury

The cerebrospinal fluid samples were centrifuged, and the catalytic activities of aspartate aminotransferase (AST) were determined by the IFCC method on a Cobas 6000 analyzer (Roche Diagnostics, Basel, Switzerland). Catalytic activities of AST in CSF exceeding 18.0 IU/L were identified as evidence of CNS tissue damage [13].

### 2.4. Statistical Methods

Concentrations of total protein, numbers of leukocytes, the percentages of lymphocytes, neutrophils and monocytes and AST catalytic activities in the CSF are in box plots expressed as a median, the 1st and 3rd interquartile range and non-outlier range of values. KEB values are divided into subgroups with aerobic metabolism (>28.0), slight anaerobic metabolism (20.0 to 28.0), moderate anaerobic metabolism (10.0 to 20.0), and strong anaerobic metabolism (<10.0) in CSF in the bar graph. The nonparametric Mann-Whitney two sample tests were performed to compare each patients group with our control group. The variables were age-adjusted before testing. The 5% level was the criterion of significance.

All statistical tests were carried out using Statistica 14.0 software (StatSoft Inc., Tulsa, OK, USA).

## 3. Results

Using the Mann-Whitney two-sample test, we compared the CSF findings of our patients with CNS involvement to normal CSF findings.

### 3.1. BCB Permeability

**Figure 2:**
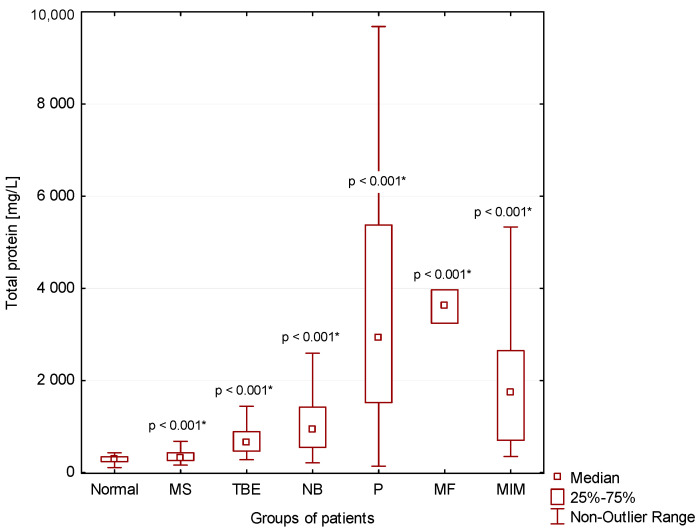
CSF total protein concentrations in our patient groups (*: statistically significant).

**Figure 3:**
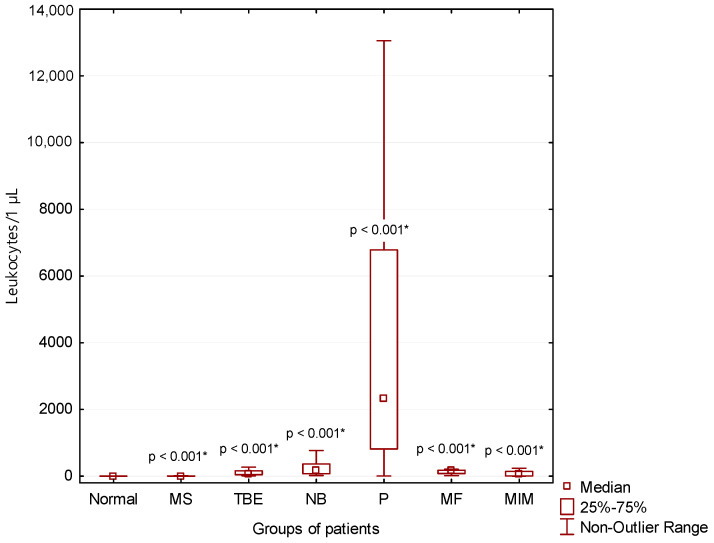
CSF leukocyte counts in our patient groups (*: statistically significant).

Compared to normal CSF findings (Normal), we found significantly higher total protein concentrations and leukocyte counts in the CSF of patients with multiple sclerosis (MS; *p* < 0.001), tick-borne encephalitis (TBE; *p* < 0.001), central neuroborreliosis (NB; *p* < 0. 001), purulent inflammation (P; *p* < 0.001), intensive inflammation with oxidative burst of macrophages (MF; *p* < 0.001), and malignant infiltration of meninges (MIM; *p* < 0.001) (Figure 2 and Figure 3).

### 3.2. Cytological Parameters

**Figure 4:**
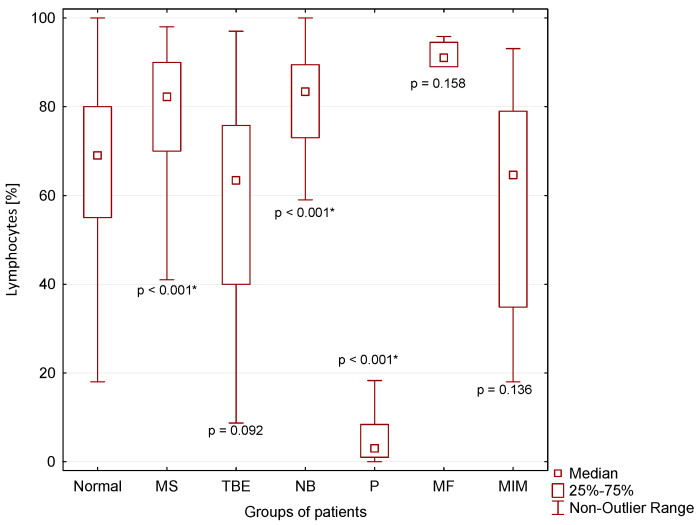
Percentage of lymphocytes in CSF in our patient groups (*: statistically significant).

A significantly higher percentage of lymphocytes in CSF compared to normal CSF findings was found in patients with MS (*p* < 0.001) and NB (*p* < 0.001), and a significantly lower percentage of lymphocytes was found in patients with purulent inflammation (P) in the CNS (*p* < 0.001 *) (Figure 4).

**Figure 5:**
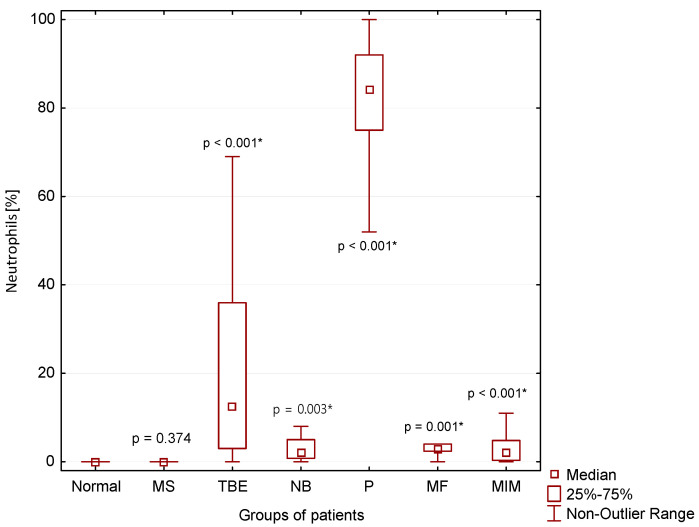
Percentage of neutrophils in CSF in our patient groups (*: statistically significant).

A significantly higher percentage of neutrophils in CSF compared to normal CSF findings was found in patients with TBE (*p* < 0.001), NB (*p* = 0.003), P (*p* < 0.001) and MIM (*p* > 0.001). The absolute highest neutrophil count is typical for patients with purulent inflammation in the CNS (P) (Figure 5).

**Figure 6:**
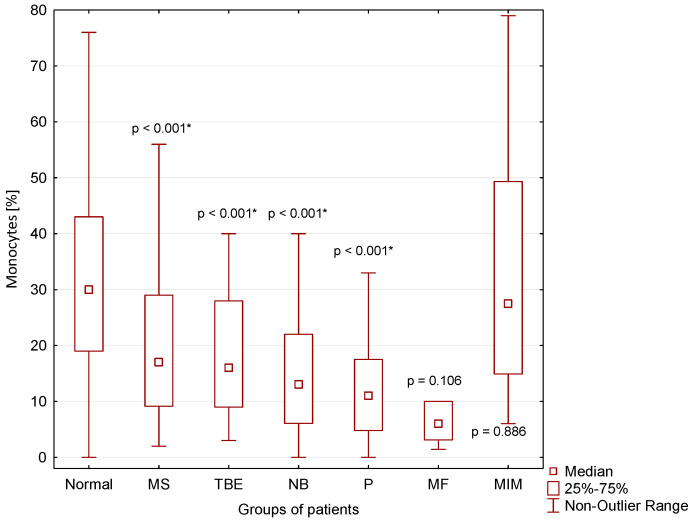
Percentage of monocytes in CSF in our patient groups (*: statistically significant).

A significantly lower percentage of monocytes in CSF compared to normal CSF findings was found in CSF of patients with MS (*p* < 0.001), TBE (*p* < 0.001), NB (*p* < 0.001) and P (*p* < 0.001) (Figure 6).

### 3.3. Energy Parameters

**Figure 7:**
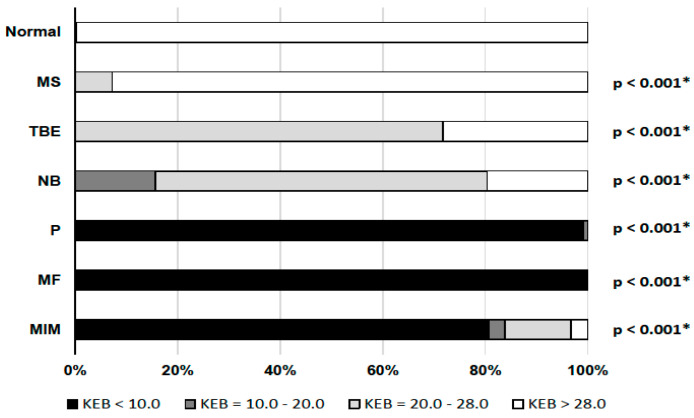
Distribution of KEB values in subgroups of our patients (*: statistically significant).

Figure 7 shows the overwhelming preponderance of cases with strongly anaerobic metabolism in the CSF (KEB < 10.0) in patients with purulent inflammation (P) and intensive inflammation with oxidative burst of macrophages (MF), and its predominance in patients with malignant infiltration of the meninges (MIM). Predominantly slight anaerobic metabolism (28.0 > KEB > 20.0) was found in CSF of patients with tick-borne encephalitis (TBE) and central neuroborreliosis (NB). We found only a few cases of mild anaerobic metabolism in CSF in patients with multiple sclerosis (MS) and 100% of cases of aerobic metabolism in CSF (KEB > 28.0) in patients with normal findings (Normal) (Figure 7).

### 3.4. Tissue Damage

**Figure 8:**
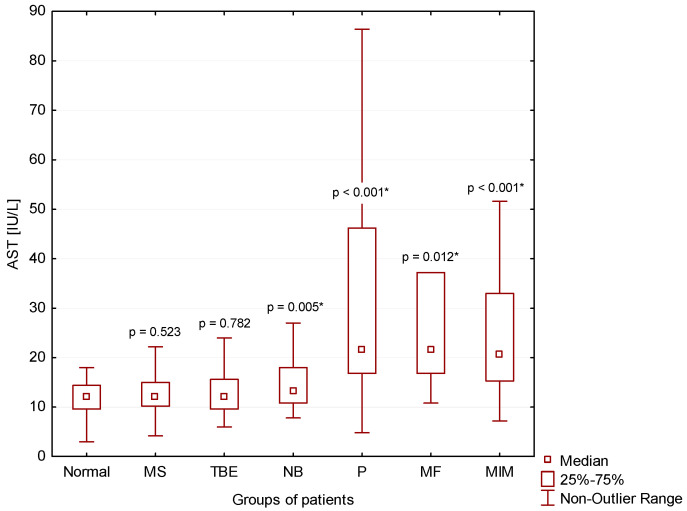
Catalytic activities of AST in CSF in our patient groups (*: statistically significant).

We found significantly higher AST catalytic activities in the CSF of patients with NB (*p* = 0.005), P (*p* < 0.001), MF (*p* = 0.012) and MIM (*p* < 0.001) compared to normal findings (Normal) (Figure 8).

## 4. Discussion

We consider basic CSF examination to be a very important part of complex CSF analysis. Its results provide key information about the current status of the CSF compartment and CNS. Many CNS impairments can be reliably detected by basic CSF analysis.

### 4.1. Inflammations in the CNS with Predominance of Lymphocytes in CSF

Lymphocytes are immunocompetent cells of adaptive immunity. Consistent with observations of many authors, we found predominance of these cells in the CSF of patients with multiple sclerosis, thick-borne encephalitis, central neuroborreliosis, malignant infiltration of meninges and a very small group of several patients with cytologically proven “serous inflammation” and energy proven “purulent inflammation” in the CSF (Table 1, Figure 4) [10,19,29,30,31,32,35,37,40,41].

### 4.2. CNS Inflammation in Multiple Sclerosis

Energy parameters, especially KEB values, allows us to distinguish multiple sclerosis patients with predominantly aerobic metabolism in the CSF (KEB > 28.0) of patients with tick-borne encephalitis and central neuroborreliosis with predominantly slightly anaerobic metabolism in the CSF (28.0 > KEB > 20.0), and in patients with malignant infiltration of meninges or in a very small group of several patients with cytologically proven “serous inflammation” and energy proven “purulent inflammation” in the CSF with strongly anaerobic metabolism in the CSF (KEB < 10.0) (Figure 7). The same delineation can almost identically be observed when assessing BCB permeability by total protein concentration in CSF and CNS tissue destruction by catalytic activities of AST in CSF (Figure 2 and Figure 8) [8,9,12,14]. Predominantly aerobic metabolism in the CSF (92.8%) of patients with multiple sclerosis is associated with predominantly normal BCB permeability (73.9%) and absence of tissue destruction in the CNS (93.0%).

We are convinced that important cause for moderate expression of inflammatory response in the CSF of patients with multiple sclerosis is the lower contribution of BCB in the pathogenesis of this disease. Autoreactive lymphocytes are migrating from the blood into the brain parenchyma across the blood-brain barrier (BBB) (Figure 1). Autoimmune immunopathological inflammation in patients with multiple sclerosis is targeting the white matter of the brain, predominantly [4,54,55,56,57,58]. In contrast, the signs of inflammation in the CSF are marginal, only.

### 4.3. Infectious Inflammations in the CNS with Predominance of Lymphocytes in CSF

On the other hand, inflammation induced by invasion of pathogens usually manifests itself directly in the CSF. Therefore, we can observe increased BCB permeability with a higher influx of immunocompetent cells and proteins into the CSF (Figure 2 and Figure 3). There is substantial demand for glucose and oxygen as immunocompetent cells are activated. This finally leads to the development of anaerobic metabolism in the CSF [8,9,10,12,14].

Lymphocytic pleiocytosis and hyperproteinorhachia are typical in patients with thick-borne encephalitis and central neuroborreliosis (Figure 2, Figure 3 and Figure 4). We found in general higher percentage of neutrophils in CSF in the early stages of tick-borne encephalitis in agreement with many authors (Figure 5) [30,34,36,38,39]. Some authors reported that the predominance of neutrophils can be confused with CSF pattern found in bacterial meningitis [34,39]. To avoid this misconduct our suggestion is to assess the energy status of CSF using KEB values. Whereas in patients with purulent inflammation induced by extracellular bacteria we found 99.1% of cases of strongly anaerobic CSF (KEB < 10.0) and 0.9% of moderately anaerobic CSF (20.0 > KEB > 10.0), in patients with tick-borne encephalitis we found 28.2% of aerobic CSF (KEB > 28.0) and 71.8% of slightly anaerobic CSF (28.0 > KEB > 20.0) (Figure 7). This is fully consistent with our previous already published results [8,9,12,14,17].

### 4.4. Purulent Inflammation in the CNS

Almost all samples of CSF of our patients with purulent inflammation in the CNS were characterized with high BCB permeability, large number of neutrophils and strong anaerobic metabolism in CSF (Figure 2, Figure 3, Figure 5 and Figure 7). These findings are in accord with the mechanism of this inflammation type. Purulent inflammation is the only inflammatory response, which cellular substrate are the cells of innate immunity, neutrophils. This type of inflammation is characterized by an oxidative burst of these cells with increased production of reactive oxygen species (ROS) [22,23,24,25,26,27,28]. The production of ROS results in significant oxygen consumption and the intensive development of anaerobic metabolism [8,9,10,12,14,15,17].

### 4.5. Infectious Inflammation with Cytologically Proven “Serous” Inflammation and Energy Proven “Purulent Inflammation”

We separately evaluated a small group of cases with cytologically proven “serous” inflammation and energy proven “purulent inflammation”. These included one patient with cryptococcal meningitis, one patient with neurotuberculosis, one patient with neurolisteriosis, and three patients with neuroborreliosis with an atypical CSF laboratory picture. These cases are very similar with regards to mononuclear pleiocytosis, strong anaerobic metabolism in the CSF compartment (KEB < 10.0) and very high BCB permeability (Table 1 and Figure 2, Figure 3, Figure 4, Figure 6 and Figure 7). Bicanic and Harrison (2005) describe the stimulation of the innate immune response through the interaction of cryptococcal mannoproteins with Toll-like receptors expressed on innate immunity cells [49]. This is followed by the activation of macrophages and their oxidative burst, which is manifested by strong anaerobic metabolism in the CSF. The similar mechanism is induced by intracellular bacteria, i.e., *Mycobacterium tuberculosis* and *Listeria monocytogenes* [62,63,64]. Our patient cohort comprised 54 confirmed cases of central neuroborreliosis. However, three of these cases showed a strikingly different CSF pattern. The high BCB permeability and strong anaerobic metabolism in the CSF compartment of these patients were more consistent with the results in patients with neurotuberculosis, neurolisteriosis, and cryptococcal meningitis (Table 1). Thus, in patients with central neuroborreliosis, a rare intensive inflammation with an oxidative burst of macrophages might also be found.

For this reason, we excluded these three cases from our group of patients with central neuroborreliosis.

### 4.6. Non-Infectious Inflammatory Response in the CSF of Patients with Malignant Infiltration of Meninges

Some authors described hyperproteinorhachia, pleiocytosis with lymphocyte predominance, and hypoglycorrhachia in the CSF of patients with malignant infiltration of meninges [51,53,59,60,61]. Our results are consistent with these findings. Marked similarity of the CSF findings in patients with neuroinfection with intracellular bacteria and yeasts allow us to speculate that an identical intensive inflammatory response with oxidative burst of macrophages is induced by tumor proliferation, in this case (Figure 2, Figure 4, Figure 6 and Figure 7).

### 4.7. AST Catalytic Activity in CSF for Assessment of CNS Parenchyma Damage

We evidenced the catalytic activity of AST in CSF as a reliable parameter to assess CNS parenchyma damage in our recent studies [8,9,18]. In this study, we found that the level of AST is correlating with the intensity of the inflammatory response expressed as anaerobic metabolism in CSF. The normal level of catalytic activity of AST in CSF (<18.0 IU/L) corresponds to samples with a predominance of aerobic, slightly anaerobic and moderately anaerobic metabolism in patients with normal CSF results, patients with multiple sclerosis or tick-borne encephalitis, respectively. Elevation of AST in CSF (>18.0 IU/L) correlated with strongly anaerobic metabolism in CSF in patients with purulent inflammation induced by extracellular bacteria and oxidative burst of macrophages induced by either intracellular bacteria or yeasts or the presence of tumor (Table 1 and Figure 7 and Figure 8).

We have recently published the significance of AST catalytic activity evaluation in CSF of patients after CNS hemorrhage [18]. Very promising results are also observed in patients with neurodegenerative CNS involvement in the long term. This could be another useful target for the measurement of this easily available and inexpensive parameter in CSF in clinical practice.

## 5. Conclusions

We consider basic CSF analysis as a solid framework for both rapid differentiation of the type of local inflammatory response in the CNS and for optimization of subsequent CSF investigation.

Inflammatory reactions in the cerebrospinal fluid are classified according to the predominant involvement of components of innate or adaptive immunity. Innate immunity activation is revealed by purulent inflammation usually induced by the presence extracellular bacteria in CNS. Reliable signs of this inflammation are extremely high numbers of neutrophils and strong anaerobic metabolism in CSF (KEB < 10.0).

Adaptive immunity activation is characterized by the presence of lymphocytes in the CSF. The significant presence of these immunocompetent cells is evident in other subgroups of our patients. Whereas aerobic (KEB > 28.0) and slight anaerobic metabolism (28.0 > KEB > 20.0) are significantly predominant in the CSF of patients with multiple sclerosis, tick-born encephalitis and central neuroborreliosis, CSF of patients with inflammation with oxidative burst of macrophages in the CNS induced by intracellular bacteria, yeasts and tumor is characterized by a predominantly strong anaerobic metabolism (KEB < 10.0).

Inflammatory response with a predominance of lymphocytes and a predominance of aerobic (multiple sclerosis) or slightly anaerobic metabolism (tick-born encephalitis and central neuroborreliosis) in the CSF is called serous inflammation. This type of inflammation is usually associated with low or slightly increased BCB permeability and absence of CNS tissue destruction. In contrast, intense inflammation with oxidative burst of neutrophils (purulent inflammation) or macrophages is associated with increased BCB permeability and destruction of CNS tissue is proven by increased catalytic activity of AST in the CSF (>18.0 IU/L).

## Figures and Tables

**Table 1:** A review of CSF analysis in several patients with neuroinfection caused by intracellular bacteria and yeasts.

	Patient 1 Cryptococcal Meningitis	Patient 2 Neurotuberculosis	Patient 3 Neurolisteriosis	Patient 4 Neuroborreliosis	Patient 5 Neuroborreliosis	Patient 6 Neuroborreliosis
Total protein [mg/L]	6926.0	3240.0	3925.0	3970.0	2060.0	3310.0
Leukocytes/1 µL	15.3	78.7	180.0	159.0	209.0	156.7
Lymphocytes [%]	15.0	94.5	95.8	89.0	90.0	92.0
Monocytes [%]	81.0	3.1	1.4	8.0	10.0	4.0
Neutrophils [%]	4.0	2.4	2.8	3.0	0.0	4.0
Glucose [mmol/L]	1.51	3.20	2.93	1.46	1.36	2.53
Lactate [mmol/L]	10.85	8.42	6.37	3.86	3.60	4.12
KEB	−91.34	−9.36	−1.13	−9.59	−9.65	8.69
AST [IU/L]	256.8	16.8	37.2	10.8	not tested	21.6

## Data Availability

All data used are with the author.
